# Coordinated distributed model predictive control for multi energy carrier systems

**DOI:** 10.1038/s41598-024-78314-5

**Published:** 2024-11-12

**Authors:** Magda I. El-Afifi, Abdelfattah A. Eladl, Magdi M. El-Saadawi, Bishoy E. Sedhom, Samaa F. Osman

**Affiliations:** 1 Electrical Eng. Deparment, Faculty of Engineering, Mansoura University, El-Mansoura, Egypt; 2 Nile Higher Institute of Engineering and Technology, El-Mansoura, Egypt

**Keywords:** Multi-energy systems, Energy hubs, Combined heat and power, Heat pump, Model predictive control, Electrical and electronic engineering, Solar energy

## Abstract

Introducing Energy hubs (EHs) is a beneficial strategy for incorporating quickly expanding renewable energies. However, the stochastic nature of renewable energy sources (RESs) and fluctuating energy demand have produced a number of difficulties, including unstable voltage/frequency, challenging energy management, and difficult network interaction. Additionally, the changing in response time of electrical and heat demands will make control challenging. This paper proposes a distributed control system for use with dynamic EHs. The RESs and loads present in the multi-carrier system cause the dynamics considering here. In order to optimise system performance, this research suggests a distributed model predictive control strategy that considers expected behaviour and operational restrictions. The strategy’s potential is demonstrated via simulations in which the proposed scheme is applied to a benchmark system.

## Introduction

Traditional energy sources can no longer meet sustainable expectations due to environmental damage and load requirement growth^1^. Renewable energy sources (RESs) have received great marks for transforming the traditional energy system^2,3^. Because RESs are geographically and climatically sensitive, it is necessary to have a highly versatile and adaptable energy supply system^4^. Energy hubs (EHs) have been proposed as a viable remedy in this regard^5–8^. The coordinated regulation of a standalone, combined heat and power generation (CHP) that is integrated with EH will be the main topic of this paper.

The most popular method for EH energy management regarding the grid’s power scheduling is a hierarchical structure^9^. The lower layer follows the load instruction, while the top layer adheres to optimal scheduling. Decentralized control structures are frequently used for the lowest layer^10^. Decentralized control, particularly for EHs with CHP, does not consider coupling. On the other hand, coupled systems’ interconnections can all be managed by a centralised control structure. Centralized control uses greater computational resources simultaneously. Centralized control can no longer handle a high-dimensional, big-data, EH optimization problem due to scale expansion and capacity upgrades.

A distributed control structure appears to hold promise for solving these problems. Although distributed control structures are dispersed as well, each controller’s control model comprises all the interrelated data from the entire system. Distributed model predictive control has been implemented in power system control in addition to model predictive control (MPC).

For the optimal power dispatch issue in systems with multiple energy carriers, Geidl and Andersson developed a model in their EH concept, which served as a starting point^11^. The matrix model and dispatch factors created by these authors were used to develop an optimization framework for the aforementioned optimal power dispatch problem. The issue of optimum power flow between diverse energy infrastructures, including district heating networks, electricity grids, and natural gas networks, has also been developed by the same authors^12^ in the context of interconnected EHs related to multi-carrier energy systems. Additionally, they developed a design optimization strategy in^13^ to obtain the ideal coupling matrix in accordance with a predetermined demand and objective function. Time-dependent factors were not taken into account because these models were presented in steady-state conditions. Then, they offered a modelling framework in^14^ that allows for the integration of an unrestricted number of energy carriers as well as chemical reactants and products. They also considered the temporal dependency, which has been highlighted in various real-world cases and included energy storage technologies in the model. A centralised model predictive controller was presented by^15^ after developing the EH idea. The aforementioned controller considers the system’s dynamics and limits while also adapting to anticipated, forecasted changes in load and energy prices. Moreover, simulations are shown applying the suggested scheme to a benchmark system with three interconnected hubs. Additionally, the effectiveness of a number of prediction horizons with various lengths has been contrasted. While the overall operation cost declines when the forecast horizon is extended, the computing effort also rises. A distributed MPC strategy was suggested in^16^ for solving the overall optimisation problem in a distributed manner (to alleviate this computing strain). To ensure that employing the control technique on a real system yields workable solutions, they have examined the performance of intermediate solutions attained through iterations using the suggested approach in a simulated case study. Additionally, cooperative behaviour was discovered, in which nearby agents support the system-wide aim. The regulation of EH outputs and internal devices is part of a dual control of EH that considers economic efficiency and security operation^17^. A dual-decomposition-based distributed algorithm was developed in^18^ to handle the problem of data and information secrecy for EH based on a consensus approach. Predictive control solutions are consistent with the EH modelling methodology is proposed in^19^ to reduce economic costs. In^20^, the authors examine how several MPC techniques work with energy management systems in buildings and EHs. Ref.^21^ presented a novel framework for distributed multi-horizon model predictive control (DMH-MPC) applied to a network of EHs. The proposed approach leverages the advantages of both distributed control and multi-horizon optimization to effectively coordinate the operation of energy hubs while considering their interactions with the broader energy grid. In^22^, a novel hybrid droop control strategy for DC microgrids that simultaneously considers operating costs and flexibility was presented. The proposed approach integrates traditional droop control with economic dispatch and flexibility assessment, offering a comprehensive solution for optimizing microgrid operation. Authors in^23^ presented a comprehensive techno-economic assessment of energy storage systems (ESSs) in multi-energy microgrids, utilizing a decomposition methodology. The proposed framework provides valuable insights into the optimal sizing, technology selection, and operational strategies for ESSs in such complex systems.

Table 1 summarises earlier literature, which has led to numerous adaptations of this framework toward an optimal power flow general, unit commitment, and demand-side management for the multi-energy management problem.

**Table 1:** Literature review summary.

Ref	Year	Problem	Goal	Approach	Architecture
^11^	2005	Modelling	Unit commitment	n.a	n.a
^12^	2005	Modelling	OPF	n.a	n.a
^13^	2005	Design	TCO	n.a	n.a
^15^	2009	Control	Unit commitment	MPC	Centralised
^16^	2010	Control	Unit commitment	MPC	Distributed
^17^	2019	Control	Unit commitment	n.a	Distributed
^11^	2021	Model	Unit commitment	Consensus	Distributed
^19^	2020	Power dispatch	Unit commitment	MPC	Centralised
^20^	2022	Energy management	Unit commitment	MPC	Centralised Distributed
This work		Control	Unit commitment	MPC	Distributed

MES = multi-energy system; OPF = optimal power flow.

Most of the researches for EH focused on the modelling and optimal operation of EH. On the other hand, few studies considered EH’s control and security operation. As a multi-carrier energy system’s most basic and significant requirement, the security operation can be achieved by regulating the EH working at a secure range with proportional power sharing. This paper proposes a distributed control system for EHs considering RESs and loads presented in the multi-carrier system. The contribution of this paper can be expressed as follow:

- The MPC framework is designed to adapt to changes in system dynamics by identifying models of inner devices based on real-time input-output data.
- The control strategy is decentralized, enabling each inner device to operate autonomously without requiring extensive communication with other devices.
- The MPC helps to maintain the security and reliability of the EH system by proactively addressing disturbances and optimizing energy allocation.
- The control approach ensures that electricity and heat outputs are proportionally allocated based on the specific needs and constraints of the EH system.

“System profile and modelling” section of this research develops a thorough theoretical model of a standalone EH for home usage. By simulating the model, the dynamic properties of such a system are determined. Then, in “Design of the control system” section, the system control structure is created. The results of the control simulation are discussed in “Control simulation” section. The paper is concluded in “Conclusions” section.

## System profile and modelling

Researchers in Power Systems and the ETH Zurich High Voltage Laboratory presented the concept of EH under a project, namely Vision of Future Energy Networks. The project aims to propose a visualization of energy systems in the future, over a long term of 20–30 years, by using the technique of Greenfield^24^. The main features of this project can be outlined as follows^25,26^:

- Moving towards MESs to take advantage of synergies between various energy carriers.
- Moving to non-hierarchical structures.
- Moving directly to interconnected and integrated energy systems.
- Joint transport of various energy carriers through longer distances in single transmitters.
- Developing EH concept: an integrated unit converts and stores multi-energy carriers.

**Fig. 1:**
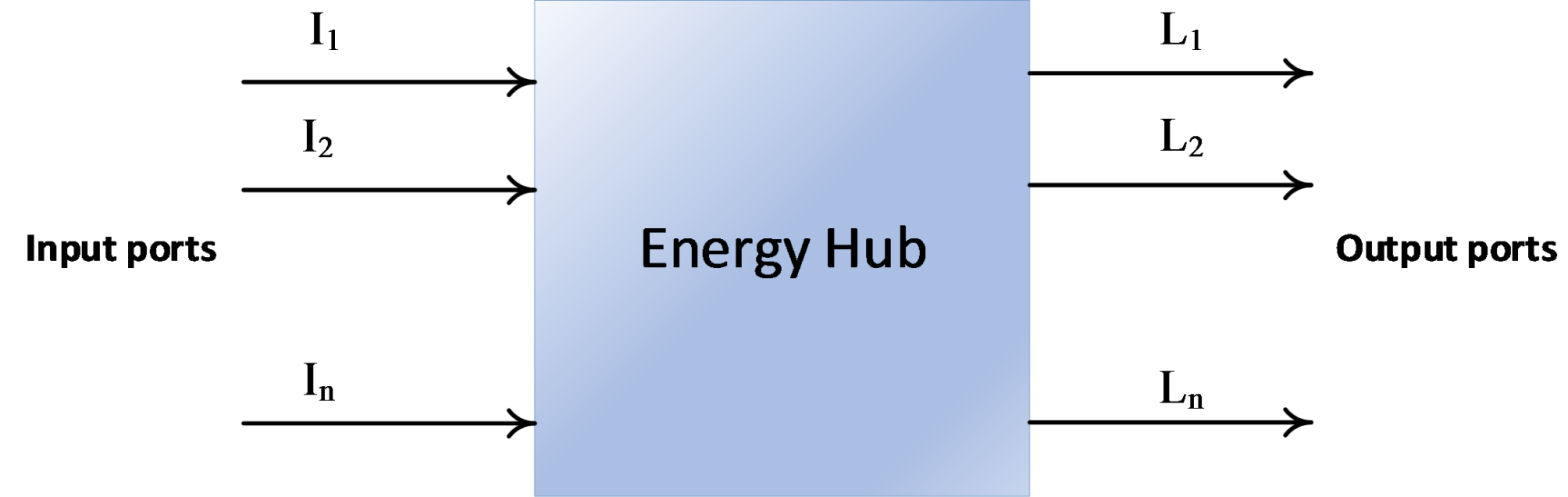
The power transformation modeling through an EH.

The EH concept was presented in^27^ as integrating ESSs, transmitters, generation, and consumers in various ways. This integration is done through converting devices or directly by dealing with single or multiple energy carriers. Figure 1 illustrates matrix model of EH and the connection of different input and output energy vectors across the coupling matrix.

Each element of the matrix defines the internal characteristics of the EH, which contain converters and conversion efficiencies for the internal components of the EH. Geidl and Andersson (2005) presented a more accurate definition of EH as follows: “EH is a framework which provides input, output, ESSs and conversion for several energy carriers”^28,29^. EHs can interface between network participants (generation and consumers) and energy infrastructure or among various energy infrastructures, such as district heat, NG, and electricity systems. The hybrid word is sometimes used with the EH, which reflects the interaction among various energy carriers in EH^30^. Thus, the EH can be defined, in brief, as: “The place where different energy carriers are received, then converted, stored and consumed”.

**Fig. 2:**
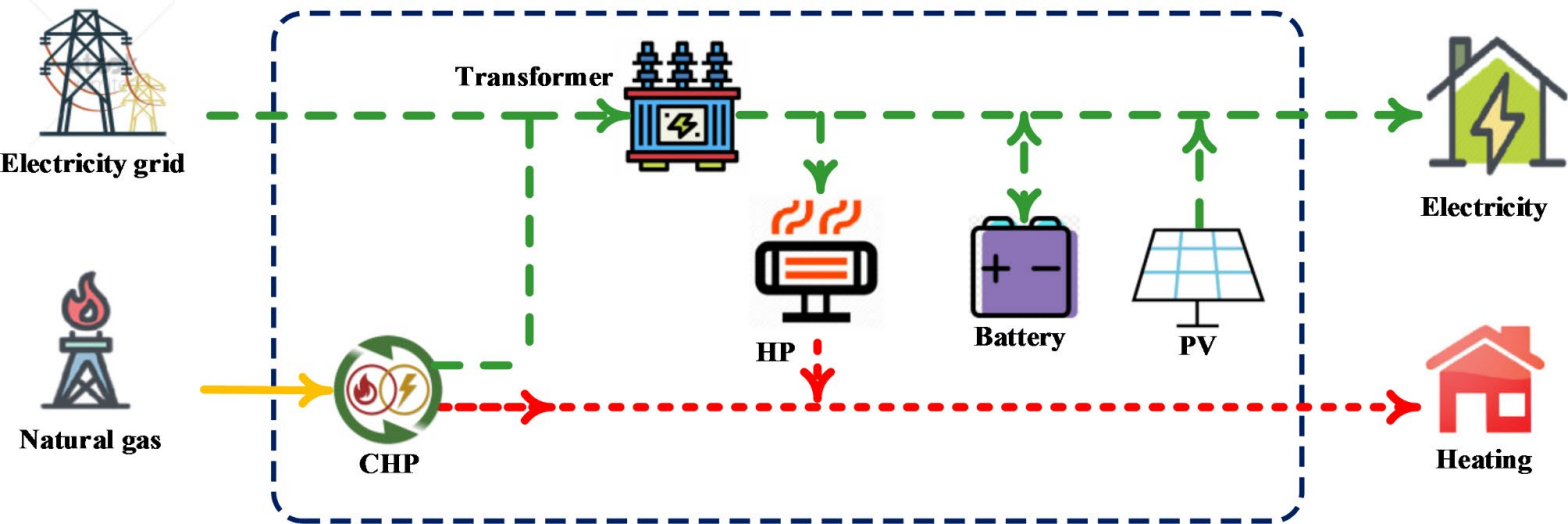
The architecture of studied EH.

A projected EH’s architecture is shown in Fig. 2. Two connected networks make up the suggested EH. Natural gas is the hub’s primary energy source, while the output provides heat and electricity. These networks are integrated through the actions of EH’s parts like CHP and EHP. The mathematical connections between the EH input and output are defined in (1) and (2) as follows^7^:

1 $$\left[L\right]=\left[C\right]\left[I\right]$$

2 $$\left[L\right]={\left[\begin{array}{c}{L}_{1}\\ \begin{array}{c}{L}_{2}\\ \vdots \\ {L}_{M}\end{array}\end{array}\right]}_{m\times 1} ,\left[C\right]={\left[\begin{array}{ccc}{C}_{11}& \dots & {C}_{1n}\\ \vdots & \ddots & \vdots \\ {C}_{m1}& \dots & {C}_{mn}\end{array}\right]}_{m\times n} ,\left[I\right]={\left[\begin{array}{c}{I}_{1}\\ \begin{array}{c}{I}_{2}\\ \vdots \\ {I}_{n}\end{array}\end{array}\right]}_{n\times 1}$$

where $L, C,$ and $I$ are the input energy carriers, coupling matrix, and output loads, respectively. These terms are further explained in the following subsections.

### System description and configuration

**Fig. 3:**
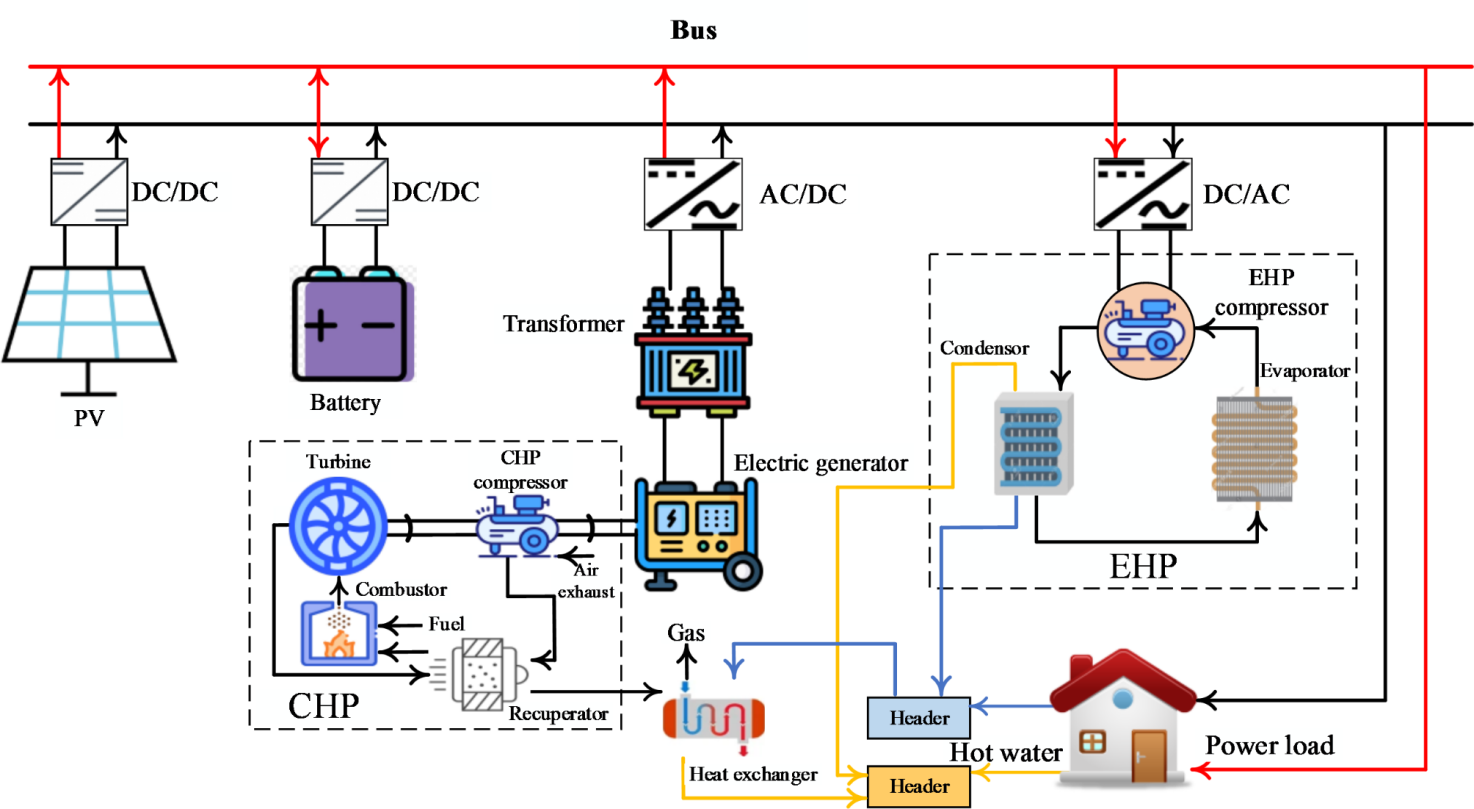
The EH system’s schematic diagram under study.

Figure 3 shows the evaluated EH’s schematic design, including CHP, photovoltaic (PV), battery, and electric heat pump (EHP). This system is designed to provide heat and electricity for home use. The PV module converts solar energy into environmentally friendly power by absorbing it. The CHP provides steady heat and electricity. The battery is essential for maintaining the grid’s voltage and for storing energy. A single CHP is insufficient to achieve the goal of concurrently meeting the power and heat demand. In this instance, the CHP and the EHP work together to meet the need for heating and hot water. The investment and energy efficiency cost determines the system’s capacity allocation. A 60kW PV module, an 80kW CHP unit, and a 60Ah battery are the allocations chosen based on a reasonable capacity proportion. Users’ thermal demands determine the EHP’s rated input power of 60kW. These sources and storage create a 380V DC microgrid. This system uses a single bus construction because of its simplicity and relatively low investment cost.

#### PV model

The PV cell, MPPT controller, and boost circuit are the three components that make up the PV module’s theoretical model. The PV cell’s I-V characteristic can be represented as^31^.

3 $${I}_{PV}={I}_{ph}-{I}_{0}\left[ exp(\frac{q}{A.K.T}(V+{R}_{S}I)-1\right]-\frac{V+{I}_{PV}{R}_{s}}{{R}_{sh}}$$

where ${I}_{PV}$ is the PV current (A), and V is the output voltage (V), ${I}_{0}$ is the PV diode reverse saturation current (A), ${I}_{ph}$ is the photo-generated current (A), ${R}_{S}$ is the series resistance (Ω), $q$ is the electric charge (C), $K$ the Boltzmann constant, $T$ is the temperature (°K), $A$ is the ideality factor, and ${R}_{sh}$ is the series resistance (Ω).

#### Combined heat and power model

The CHP system comprises a centrifugal compressor, a radial turbine, a combustion chamber, and a recuperator. Similar to the fully regenerative CHP described in this paper, the turbine’s high-temperature exhaust gas heats all of the compressed air. A modular modelling approach is utilised due to the CHP components’ high degree of independence^32^. Each component’s fundamental formulas are listed below.

The best way to determine the compressor’s precise performance is typically by experimentation, which may be condensed into a set of equations^33^.

4 $${T}_{c2}={T}_{c1}\left[1+\left({\pi }_{c}^{{k}_{a}}-1\right)\right]/{\eta }_{c}$$

5 $${P}_{c}={m}_{c}{c}_{pa}{T}_{c1}\left({\pi }_{c}^{{k}_{a}}-1\right)/{\eta }_{c}$$

where ${T}_{c1}$ and ${T}_{c2}$ are inlet and outlet temperatures of the compressor (°C), ${k}_{a}$ is the air adiabatic index, ${P}_{c}$ is the power of the compressor (kW), ${m}_{c}$ is the mass flow rate of the compressor (kg/s), ${c}_{pa}$ is the air specific heat capacity (kJ/(kg. °C)), and $\pi_{c} /\eta_{c}$ is reduced parameters of specific compressors.

Calculations for the outlet flow rate of the combustor, temperature, and pressure include can be formulated as

6 $${m}_{b1}=\left(1-{k}_{cl}\right){m}_{c},{m}_{b2}={m}_{b1}+{m}_{f}$$

7 $${T}_{b2}=\left[{m}_{b1}{c}_{pa}{T}_{c2}+{m}_{f}\left({Q}_{cv,f}{\eta }_{b}+{i}_{f}\right)\right]/{c}_{pg}{m}_{b2}$$

8 $${p}_{b2}={\varepsilon }_{b}{p}_{b1}$$

where ${m}_{b1,}{m}_{b2}$ are the inlet and outlet mass flow rate of the combustor (kg/s), ${k}_{cl}$ is the air-cooling coefficient, ${m}_{f}$ is the fuel mass flow rate (kg/s), ${T}_{b2}$ is the outlet temperature of the combustor (°C), ${Q}_{cv,f}$ is the calorific fuel value (kJ/kg), ${i}_{f}$ is the physical fuel enthalpy (kJ/kg), ${c}_{pg}$ is the combustor specific heat capacity (kJ/(kg. °C)), and ${p}_{b1},{p}_{b2}$ are the inlet and outlet flow pressure of the combustor (kPa).

The reduced parameters ${\pi }_{t}$ and ${\eta }_{t}$ are necessary for turbine modelling. When the gas pipeline’s heat exchange among the combustor and the turbine is ignored, the inlet temperature of the turbine can be taken to be the same as the combustor output temperature. Consequently, it is possible to calculate the turbine’s power and outlet temperature using

9 $${T}_{t1}={T}_{b2}$$

10 $${T}_{t2}={T}_{t1}\left[1\left(1-1/{\pi }_{t}^{{k}_{g}}\right)/{\eta }_{t}\right]$$

11 $${P}_{t}={m}_{t}{c}_{pa}{T}_{t1}\left(1-1/{\pi }_{t}^{{k}_{a}}\right)/{\eta }_{t}$$

where ${T}_{t1}$ and ${T}_{t2}$ are the inlet and outlet temperature of the turbine (°C), $\pi_{t} /\eta_{t}$ is reduced parameters of the specific turbine $s, {k}_{g}$ is the gas adiabatic index, ${P}_{t}$ is the power of the turbine (kW), and ${m}_{t}$ is the turbine mass flow rate (kg/s).

Due to its high transfer efficiency and compactness, main surface heat transfer is the most popular form of recuperator for a CHP system. The air and exhaust gas heat transfer process are best described as^33^:

12 $$\left\{\begin{array}{c}\frac{\partial \left({\rho }_{a}{A}_{a}{c}_{pa}{T}_{a}\right)}{\partial t}+\frac{\partial \left({m}_{a}{c}_{pa}{T}_{a}\right)}{\partial x}+{\alpha }_{a}{A}_{a}\left({T}_{a}-{T}_{j}\right)=0\\ \frac{\partial \left({\rho }_{g}{A}_{g}{c}_{pg}{T}_{g}\right)}{\partial t}+\frac{\partial \left({m}_{g}{c}_{pg}{T}_{g}\right)}{\partial x}+{\alpha }_{g}{A}_{g}\left({T}_{g}-{T}_{j}\right)=0\\ {\rho }_{j}{c}_{pj}{A}_{j}\frac{\partial {T}_{j}}{\partial \tau }+{\alpha }_{g}{A}_{g}\left({T}_{g}-{T}_{j}\right)+{\alpha }_{a}{A}_{a}\left({T}_{a}-{T}_{j}\right)=0\end{array}\right.$$

where ${\rho }_{a},{\rho }_{g},{\rho }_{j}$ are the air, gas and metal wall density (kg/m^3^), ${A}_{a},{A}_{g},{A}_{j}$ are the air, gas and metal wall heat transfer area (m^2^), ${T}_{a},{T}_{g}$ are the air and gas temperature (°C), ${m}_{a},{m}_{g}$ is the air and gas mass flow rate (kg/s), ${\alpha }_{a},{\alpha }_{g}$ are the air and gas heat transfer coefficient (W/m^2^.K), ${T}_{j},{T}_{g}$ are the metal wall and gas temperature (°C) and ${c}_{pj}$ is the metal wall-specific heat capacity (kJ/(kg. °C)).

The rotor’s dynamic model is created as:

13 $$J\omega \frac{d\omega }{d\tau }={P}_{t}-{P}_{c}-{P}_{fr}-{P}_{e}$$

14 $${P}_{e}={P}_{ed}{\left(n/{n}_{d}\right)}^{2}$$

where ${P}_{c},{P}_{fr},{P}_{e}$ are the compressor, mechanical friction loss and rotor power (kW), ${P}_{ed}$ is the rated rotor power (kW), $n$ is the polytropic exponent and ${n}_{d}$ is the rated rotational speed of the turbine (m/s).

The waste heat from the exhaust gas is utilised in a heat exchanger. The heat transfer function is given as, presuming that the feed water’s temperature is constant.

15 $$\varnothing ={m}_{g}{c}_{pg}\left({T}_{g1}-{T}_{g2}\right)={m}_{a}{c}_{pa}\left({T}_{a2}-{T}_{a1}\right)$$

16 $$\varnothing =\alpha A\left[\left({T}_{g1}-{T}_{a2}\right)-\left({T}_{g2}-{T}_{a1}\right)\right]/ln\frac{{T}_{g1}-{T}_{a2}}{{T}_{g2}-{T}_{a1}}$$

where ${T}_{g1}{,T}_{g2}$* i*s the inlet and outlet temperature of the exhaust gas (°C), ${T}_{a1}{,T}_{a2}$* i*s the inlet and outlet temperature of the air (°C), $\varnothing$ is the heat exchange capacity (kW).

#### Battery model

Among the most popular models of batteries are mathematical models and electrical equivalent circuit network models electrochemical models. This article mostly ignores the physicochemical mechanism and concentrates instead on the process of charging/discharging and the relationships among voltages and currents^34^. The battery swiftly transitions between the charging and discharging states to equipoise the energy supply and demand quickly. Thus, a bidirectional DC/DC converter connects the battery to the grid. When the supply and demand for electricity are out of balance, the battery will start working right away to stabilise the bus voltage within a second and prevent the other loads from operating abnormally due to bus voltage fluctuations.

#### EHP model

An EHP system’s major parts are its compressor, condenser, evaporator, and expansion valve. The mathematical model of EHP will be provided in a modular format due to each component’s independence. Temperature modelling uses the lumped parameter approach for simplicity^35^. The evaporator’s thermal balance equation may be created using the law of conservation of energy by assuming that the flow rate in the evaporator is equal to that in the compressor and disregarded pressure and heat loss through evaporation, which can give as follows:

17 $$\frac{1}{2}{M}_{w,ev}{c}_{pw}\frac{d{t}_{w1,ev}+d{t}_{w2,ev}}{d\tau }={m}_{ev}{c}_{pw}\left({T}_{w1,ev}-{T}_{w2,ev}\right)-{Q}_{ev}$$

18 $${M}_{r,ev}\frac{d{h}_{r,ev}}{d\tau }={Q}_{r,ev}-{m}_{r,ev}\left({h}_{1}-{h}_{4}\right)$$

19 $${Q}_{ev}={\alpha }_{ev}{A}_{ev}\left[\left({t}_{w1,ev}-{t}_{w2,ev}\right)/2-{t}_{ev}\right]$$

where ${M}_{w,ev}$ is the evaporated water quantity (kg), ${c}_{pw}$ is the water-specific heat capacity (kJ/(kg. °C)), ${t}_{w1,ev},{t}_{w2,ev}$* i*s the inlet and outlet temperature of evaporated water (°C), ${m}_{ev}$ is the evaporated water mass flow rate (kg/s), ${Q}_{ev}$ is the heat capacity of evaporated water (kW), ${\alpha }_{ev}$ is the evaporated water heat transfer coefficient (W/m^2^.K), and ${A}_{ev}$ is the evaporated water heat transfer area (m^2^).

The performance test data of the particular compressor type are the foundation for the compressor model. The crucial variable that captures the compressor’s characteristics is the pressure ratio between condensing and evaporating pressures, or $\varepsilon ={P}_{de}/{P}_{ev}$,, where ${P}_{de}$ and ${P}_{ev}$ depend on the condenser and evaporated water properties, respectively. So, it is possible to determine the compressor outlet refrigerant enthalpy and its power requirement as^33^:

20 $${m}_{r}={V}_{cp}{\omega }_{cp}{\eta }_{v,cp}{\lambda }_{v,cp}/{v}_{r1}$$

21 $${h}_{2}={h}_{1}+{P}_{c}/{m}_{r,cp}$$

22 $${P}_{c}={P}_{t}/{\eta }_{e,eff}=\frac{{n}_{cp}}{1-{n}_{cp}}{\eta }_{v,cp}{V}_{cp}{\omega }_{cp}{p}_{e}\left[1-{\varepsilon }^{\left({n}_{cp}-1\right)/{n}_{cp}}\right]$$

where ${m}_{r}$ is the refrigerant water mass flow rate (kg/s), ${V}_{cp}$ is the gas displacement of the EHP compressor (m^3^), ${\omega }_{cp}$ is the rotational speed of the EHP compressor (r/s), ${\eta }_{v,cp}$ is the volume efficiency of the EHP compressor, ${v}_{r1}$ is the specific volume of refrigerant water (m^3^/kg), ${h}_{1},{h}_{2}$ are the inlet and outlet enthalpy of the compressor (kJ/kg), ${m}_{r,cp}$ is the refrigerant water of EHP compressor mass flow rate (kg/s), ${\eta }_{e,eff}$ is the electrical efficiency, and ${n}_{cp}$ is the polytropic exponent of the EHP compressor.

Condenser operation is actually the exact opposite of evaporator operation. Consequently, the model may be written as^33^:

23 $$\frac{1}{2}{M}_{w,de}{c}_{pw}\frac{d{t}_{w1,de}+d{t}_{w2,de}}{d\tau }={Q}_{de}-{m}_{de}{c}_{de}\left({T}_{w2,de}-{T}_{w1,de}\right)$$

24 $${M}_{r,de}\frac{d{h}_{r,de}}{d\tau }={m}_{r,de}\left({h}_{1}-{h}_{4}\right)-{Q}_{r,de}$$

25 $${Q}_{de}={\alpha }_{de}{A}_{de}\left[{t}_{de}-\left({t}_{w1,de}-{t}_{w2,de}\right)/2\right]$$

Where ${M}_{w,de}$ is the condenser water quantity (kg), ${t}_{w1,de},{t}_{w2,de}$* i*s the inlet and outlet temperature of condenser water (°C), ${m}_{de}$ is the condenser water mass flow rate (kg/s), ${c}_{de}$ is the condenser water-specific heat capacity (kJ/(kg. °C)), ${Q}_{de}$ is the heat capacity of condenser water (kW), ${\alpha }_{de}$ is the condenser water heat transfer coefficient (W/m^2^.K), and ${A}_{de}$ is the condenser water heat transfer area (m^2^).

Before and after going through the expanding valve, the enthalpy value is the same, if the refrigerant flow rate stays constant, namely$, {h}_{3}={h}_{4}$

#### Header model

Before being delivered to home heating, the two pathways of hot water produced by the CHP and the EHP are combined in a header. The mixture’s hot water temperature is computed as^33^:

26 $$Q={c}_{pa}{m}_{hw,CHP}\left({t}_{hw}-{t}_{hw,CHP}\right)={c}_{pa}{m}_{hw,EHP}\left({t}_{hw,EHP-}{t}_{hw}\right)$$

27 $${t}_{hw}=\frac{{m}_{hw,EHP}{t}_{hw,EHP}+{m}_{hw,CHP}{t}_{hw,CHP}}{{m}_{hw,EHP}+{m}_{hw,CHP}}$$

where ${m}_{hw,CHP}, {m}_{hw,EHP}$ are the CHP and EHP hot water mass flow rate (kg/s).

### Analysis of dynamic characteristics

A virtual representation of the real system using a theoretical model was created. This allows us to analyze the system’s overall open-loop dynamic behavior. While the system involves many variables, only a few are adjustable: feed water flowrate, EHP compressor rotating speed, and CHP fuel flowrate. These primary controllable variables must maintain a balanced energy supply and demand by regulating indoor room temperature, feed water temperature, and the difference between EHP power demand and CHP power supply.

**Fig. 4:**
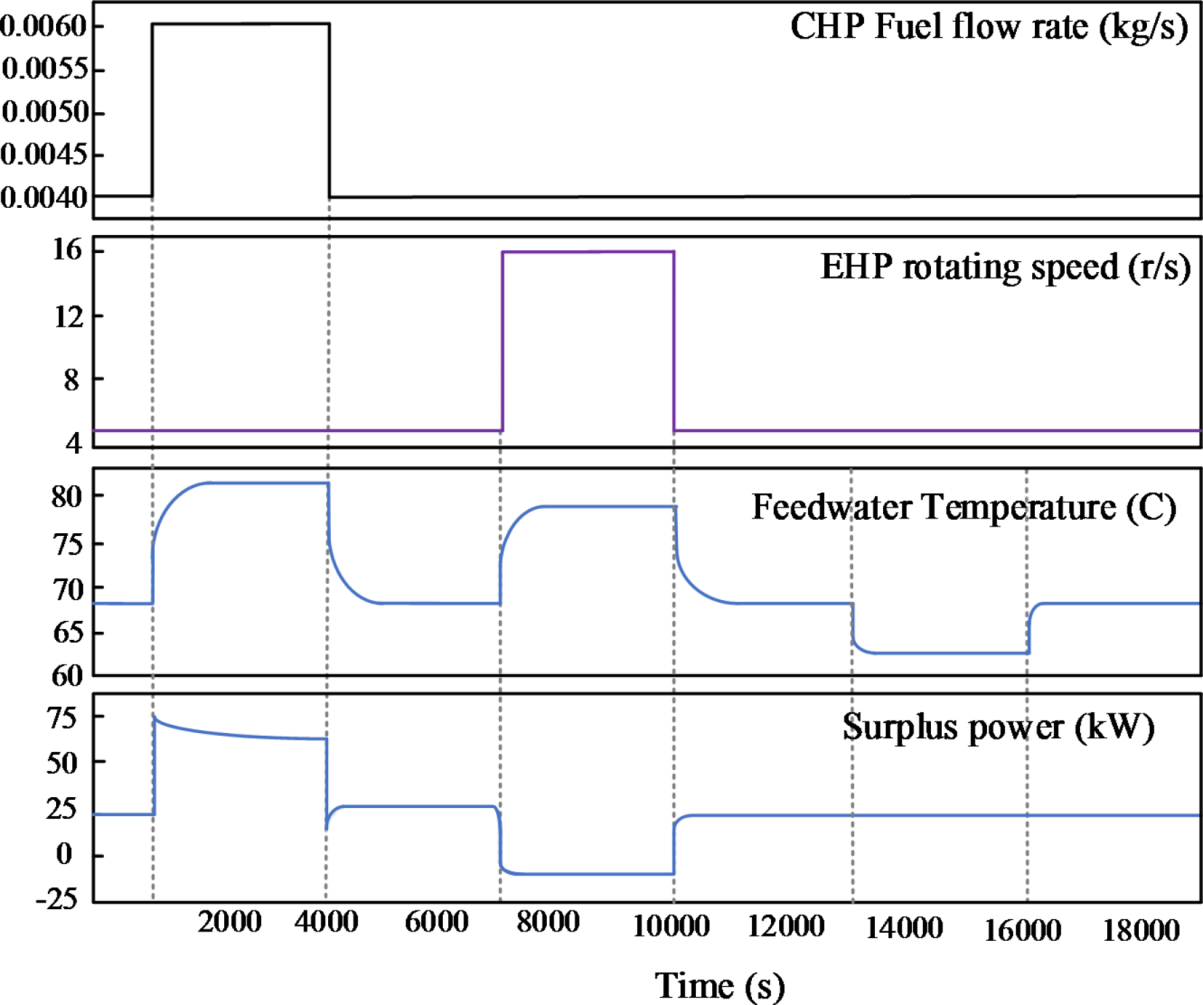
Open-loop step responses of controlled variables.

Based on the previous analysis, Fig. 4 represents the controlled variables’ open-loop step reaction to the stepped adjustable variables. The flowrate of fuel of CHP rises from 0.004 kg/s to 0.006 kg/s at 1000s and then falls to its initial value at 4000s. The EHP compressor’s rotational speed increases from 5 r/s to 16 r/s at 7000s and then recovers at 10,000 seconds. Each controlled variable’s reasonable transient processes and tendencies show the model’s realism. The response data clearly distinguishes between electrical and thermal processes’ dynamic properties. Heat production takes longer to attain equilibrium than electricity production. The open-loop response offers a fundamental understanding of the dynamic nature of the CHP-EHP. It also establishes the framework for analysing the difficulties in designing the control system.

## Design of the control system

The challenges in control are suggested in this part depending on a study of the dynamic properties of the entire system. Two types of issues plague the real-time coordinated control of the system. The necessity for the control system’s disturbance rejection capability is brought on by the uncertain nature of PV power output and the unpredictability of user demand. Day-ahead EH optimum scheduling frequently employs some form of stochastic forecasting methodology and rejects these types of interruptions. However, the real-time control system for the EH base layer must treat such energy variations as unknown disturbances, which puts a strain on the control system’s capacity for disturbance rejection. The tight connection and disparate dynamic properties of electrical and thermal processes hamper the coordinated control. The EH is a standalone system; therefore, all energy is produced and used inside the system. Additionally, because CHP and EHP exist, the supply of electrical and thermal energy is coupled, and if one side of the processes is out of balance, the other will follow. Due to these difficulties, the control system must be able to work well during disturbances and handle connections. A workable control system structure to solve these issues is provided in the following section.

### Structure of the control system

As mentioned above, Fig. 2 produces three outputs: the temperature of the indoor room ${T}_{room}$, the temperature of the feedwater ${T}_{feed}$ and the difference among CHP power supply ${P}_{CHP}$ and EHP power demand ${P}_{EHP}$. The manipulated variables consist of the flowrate of the fuel of CHP ${m}_{f}$, EHP’s compressor’s rotating speed ${r}_{c}$, and feedwater’s flowrate ${D}_{feed}$. An index termed excess power is created to indicate the equilibrium between the supply and demand for electricity, as seen below:

28 $${P}_{s}={P}_{CHP}{-P}_{PV}{-P}_{EHP}{-P}_{user}$$

where the variables denote PV power generation and user power consumption ${P}_{PV}$ and ${P}_{user}$, respectively. ${P}_{PV}$ and ${P}_{user}$ are considered disturbances whereas ${P}_{CHP}$ and ${P}_{EHP}$ are controllable. Although the feedwater flowrate influences both controllable variables, the effect is indirect and subtle. Therefore, it makes more sense to control the ${T}_{feed}$ and surplus power ${P}_{s}$ with ${m}_{f}$ and ${r}_{c}$.

**Fig. 5:**
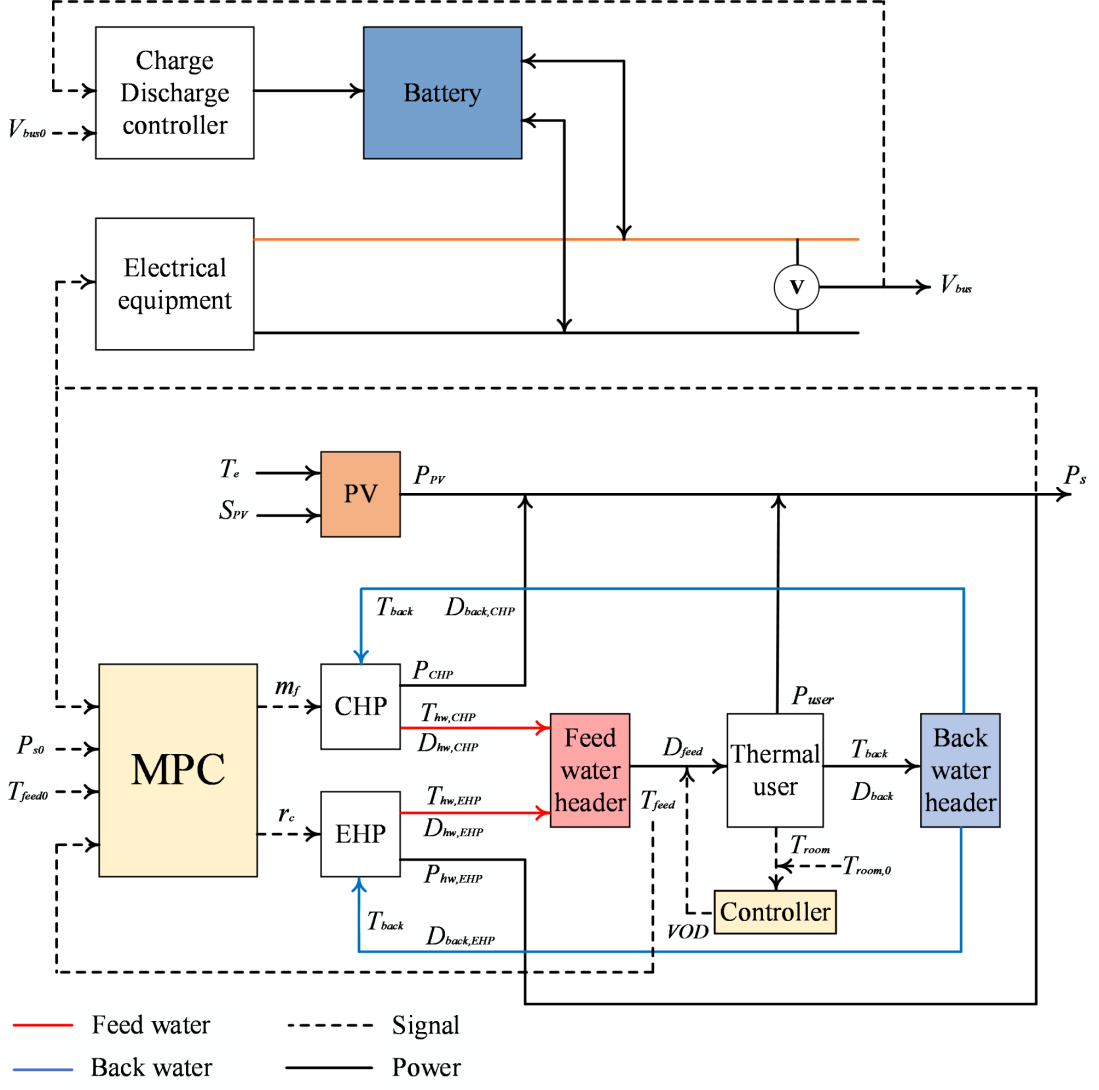
Architecture of control system.

Figure 5 shows the organisation of the control system in its entirety. Two controllers locally are present. The first is a battery charge/discharge management system that stabilizes the grid voltage ${V}_{BUS}$. Another option is to have the local PI controller adjust the feed water flow to regulate the feed water temperature. The coordinated controller is the heart of the control system. The coordinated controller collects ${T}_{feed}$ and ${P}_{s}$ real-time data and compares it to the set points. The values of ${m}_{f}$ and ${r}_{c}$ for the following control, the period will then be determined by the control algorithm. To address the difficulties of EH control, the coordinated control algorithm needs to have high disturbance-rejection and coordinated control capabilities.

### System identification

Creating a mathematical model of a system using measurement data is known as system identification^36^. The term “identification” was initially used by Zadeh^36^, to describe the issue of figuring out a black box’s or model’s input-output linkages using experimental data sets. A model of the system is required for controller design and behaviour prediction.

Two control models were presented, one by controlling the CHP unit only, and the other by controlling the two CHP-EHP units. Following are the definitions of controlled and manipulated variables: the CHP’s fuel flowrate, ${m}_{s}$, as the first manipulated variable ${u}_{1}$, the rotating speed of EHP’s compressor, ${r}_{c}$ , as the second manipulated variable ${u}_{2}$, the surplus power, ${P}_{s}$, as the first controlled variable ${y}_{1}$, and the temperature of feed water, ${T}_{feed}$, as the second controlled variable ${y}_{2}$.

The control system’s input-output pairing matrix of the CHP unit only can be shown as:

29 $$\left[\begin{array}{c}{P}_{s}\\ {T}_{feed}\end{array}\right]=\left[\begin{array}{c}{G}_{11}(S)\\ {G}_{21}(S)\end{array}\right]\left[{m}_{s}\right]=G(S)$$

where ${G}_{11}(S)$, and ${G}_{21}(S)$ denote transfer functions respectively. Before importing the step-response data into the MATLAB/System Identification Toolbox, the steady-state value was taken out. The determined transfer function matrix is provided as follows:

30 $$G(S)=\left[\begin{array}{c}{G}_{11}(S)\\ {G}_{21}(S)\end{array}\right]=\left[\begin{array}{c}\frac{-1672s+1504}{{s}^{2}+0.2179s+0.3134}\\ \frac{5767s+1556}{{{s}^{3}+0.167s}^{2}+1.891s+0.1044}\end{array}\right]$$

Consequently, the control system’s input-output pairing matrix of CHP-EHP can be shown as follows:

31 $$\left[\begin{array}{c}{P}_{s}\\ {T}_{feed}\end{array}\right]=\left[\begin{array}{cc}{G}_{11}(S)& {G}_{12}(S)\\ {G}_{21}(S)& {G}_{22}(S)\end{array}\right]\left[\begin{array}{c}{m}_{s}\\ {r}_{c}\end{array}\right]=G(S) \left[\begin{array}{c}{m}_{s}\\ {r}_{c}\end{array}\right]$$

where ${G}_{11}(S)$, ${G}_{21}(S)$, ${G}_{12}(S)$ and ${G}_{22}(S)$ denote transfer functions respectively. Before importing the step-response data into the MATLAB/System Identification Toolbox, the steady-state value was taken out. The determined transfer function matrix is provided as follows:

32 $$G\left( S \right) = \left[ {\begin{array}{*{20}c} {G_{11} \left( S \right)} & {G_{12} \left( S \right)} \\ {G_{21} \left( S \right)} & {G_{22} \left( S \right)} \\ \end{array} } \right] = \left[ {\begin{array}{*{20}l} {20590\left( {1 + 7.1013s} \right)} \hfill & { - 3.1189\left( {1 + 111.74s} \right)} \hfill \\ {\left( {1 + 4.6811s} \right)\left( {1 + 1.693s} \right)} \hfill & {\left( {1 + 121.24s} \right)\left( {1 + 0.41149s} \right)} \hfill \\ {6458.3\left( {1 + 271.41s} \right)} \hfill & {0.95459\left( {1 + 112.89s} \right)} \hfill \\ {\left( {1 + 331.83s} \right)\left( {1 + 104.04s} \right)} \hfill & {\left( {1 + 202.8s} \right)\left( {1 + 29.223s} \right)} \hfill \\ \end{array} } \right]$$

### MPC controller design

MPC is an optimal control technique in which the calculated control actions minimize a cost function for a constrained dynamical system over a finite, receding horizon.

An MPC controller receives or guesses the current status of the plant at each time step. A restricted optimization problem that depends on the current system state and an internal plant model is then solved to determine the sequence of control actions that minimise the cost over the horizon. The controller then only applies the first computed control action to the plant, ignoring the subsequent ones. The MPC controller was designed based on the discrete state space model of the CHP-EHP system.

33 $${x}_{d}(k+1)= {A}_{d}{x}_{d}(k)+{B}_{d}u(k)$$

34 $$y(k) = {C}_{d}{x}_{d}(k)$$

where is $y(k)$ is the output, $u(k)$ is the input, ${x}_{d}$ is the state, ${A}_{d}$ is the state transition matrix, ${B}_{d}$ is the input matrix multiplied by the input u(k), and ${C}_{d}$ is the output matrix.

The integral embedded augmented model is created for the subsequent controller design in order to achieve zero offsets.

35 $$\overbrace {{\left[ {\begin{array}{*{20}l} {\Delta x_{d} \left( {k + 1} \right)} \hfill \\ {y\left( {k + 1} \right)} \hfill \\ \end{array} } \right]}}^{{x\left( {k + 1} \right)}} = \overbrace {{\left[ {\begin{array}{*{20}c} {A_{d} } & O \\ {C_{d} A_{d} } & I \\ \end{array} } \right]}}^{A}\overbrace {{\left[ {\begin{array}{*{20}c} {\Delta x_{d} \left( k \right)} \\ {y\left( k \right)} \\ \end{array} } \right]}}^{x\left( k \right)} + \overbrace {{\left[ {\begin{array}{*{20}c} {B_{d} } \\ {C_{d} B_{d} } \\ \end{array} } \right]}}^{B}\Delta u\left( k \right)$$

36 $$y\left( k \right) = \overbrace {{\left[ {\begin{array}{*{20}c} O & I \\ \end{array} } \right]}}^{C}\left[ {\begin{array}{*{20}l} {\Delta x_{d} \left( k \right)} \hfill \\ {y\left( k \right)} \hfill \\ \end{array} } \right]$$

The state vector $x(k)$ is now composed of the original state ${x}_{d}(k)$ and an additional integral term ${\Delta x}_{a}(k)$. The matrix $A$ is the augmented state transition matrix, $B$ is the augmented input matrix, $\Delta u(k)$ is the change in input, and $C$ is the augmented output matrix.

The cost function and the constraints are given in (37).

37 $$min J = {\Vert {Y}_{r}-Y\Vert }_{Q}^{2}+{\Vert \Delta U\Vert }_{R}^{2}$$

38 $$s.t. {u}_{min}\le u\le {u}_{max}$$

39 $$\Delta {u}_{min}\le \Delta u\le {\Delta u}_{max}$$

## Control simulation

This section presents an application of the proposed methodology to manage an EH is presented in this section. The EH system shown in Fig. 2 is subjected to the suggested MPC technique. The simulation is performed in a MATLAB environment using the MPC Designer toolbox solver. To verify the dynamics of the model, the step disturbance is applied to each input of the model in two case studies. The case studies display the impact of integrating a distributed controller with the different units. In the first case study, MPC is integrated with the CHP unit. In order to clarify the effect of this controller on the system, an excess of electricity generation and an increase in heat demand are assumed. The other case study is configured by integrating the MPC with CHP-EHP units. An increase in both electric and heat demand is assumed as a disturbance to illustrate the effect of the controller in this case. The controller parameters are listed in Table 2.

**Table 2:** Controller paramerters.

Item	N_p_	N_c_	Q		R		Cdis	
MPC	20	1	y_1_	Y_2_	u_1_	u_2_	C_d,11_	C_d,22_
			1	50	0.2	1	50	500

N_p_: Prediction horizon, N_c_: Control horizon, Q: Weighting matrix for the output variables (y_1_ and y_2_), R: Weighting matrix for the control inputs (u_1_ and u_2_), Cdis: Diagonal matrix containing the costs associated with disturbances.

### Case study 1 (CHP)

**Fig. 6:**
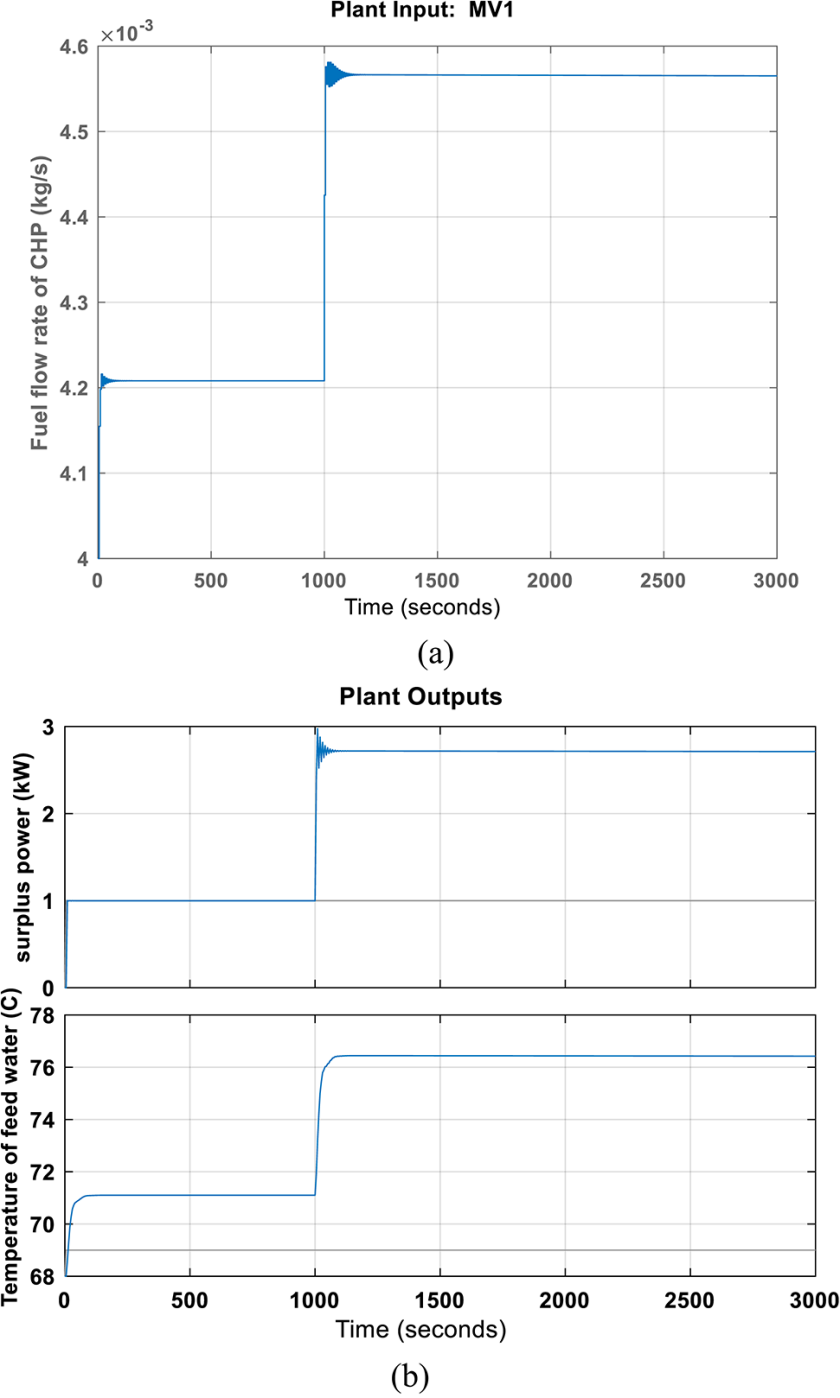
Disturbance rejection results of MPC of CHP when electrical and heat requirements increase simultaneously at 1000s.

In this case, an excess of electricity generation and increased heat demand are assumed. The MPC’s results for disturbance rejection control under simultaneous electrical and thermal disturbance are displayed in Fig. 6. As shown in the figure, the surplus power increases by 3kW due to the PV unit output at 1000s. At the same time, the heat demand increases. So, the indoor room temperature set-point rises from 68 to 76.4 °C throughout this time. To make up for the lack of power, the CHP fuel flowrate drastically rises from approximately 0.00421 kg/s to 0.00458 kg/s. The MPC took about 200 s to restore the system to a stable state.

### Case study 2 (CHP-EHP)

**Fig. 7:**
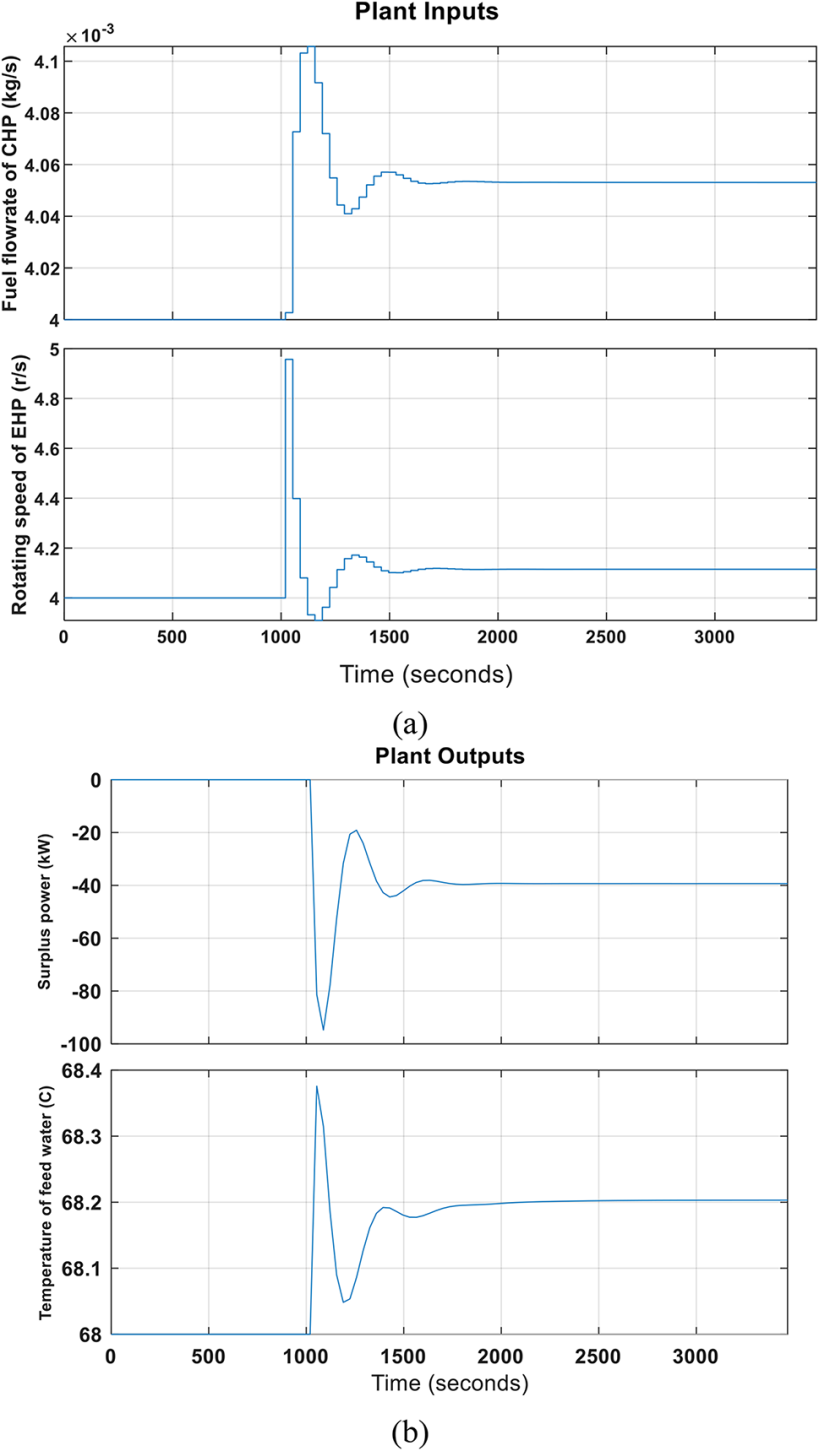
Disturbance rejection results of MPC of CHP-EHP when electrical and heat requirements increase simultaneously at 1000s.

In this case, an electric and heat demand increase is assumed. The MPC’s results for disturbance rejection control under simultaneous electrical and thermal disturbance are displayed in Fig. 7 As shown in the figure; the surplus power decreases by 95kW at 1000s while the heat demand increases. So, the indoor room temperature set-point rises from 68 to 68.4 °C throughout this time. To make up for the lack of power, the CHP fuel flowrate drastically rises from approximately 0.004 kg/s to 0.00421 kg/s. The rotational speed of the EHP’s compressor increases due to increased loads. The MPC took about 500 seconds to restore the system to a stable state.

It is clear from the abovementioned results that the MPC in the first case was faster in restoring the stable state in the system, with a 60% decrease in time compared to case 2. This is due to the use of one controller with the CHP unit (distributed control) compared to using one controller on the CHP and EHP units (centralized control).

## Conclusions

The energy supply by EH is coordinated in this work using an MPC algorithm. By developing a theoretical model, the dynamic properties of EH are investigated. Stimulating the model reveals the differences between the processes used to supply heat and power. Then, a control structure of the system is suggested based on the understanding of control difficulties. The coordinated controller is at the heart of the control system. For coordination, cooperative distributed MPC is used. The simulation results show the effectiveness of MPC, and the system’s distributed nature significantly reduces the computing load. The future trend will be the application of MPC to larger-scale, regionally interconnected EH with more varied energy source and equipment combinations. Further research will be done on the connection between real-time distributed control and optimum scheduling. The study of integrated scheduling and control further prompted the significance of the proposed control method for practical application. This allowed for consideration of the system’s actual operation mode, including shut-down and start-up.

## Data Availability

The datasets used and generated during the current study are available from the corresponding author upon reasonable request.
